# SG-APSIC1163: A five-year review and analysis of sharp injuries in an acute-care hospital in Singapore

**DOI:** 10.1017/ash.2023.59

**Published:** 2023-03-16

**Authors:** Helen Oh, Mervis Mak, Tuodi Wu

**Affiliations:** Changi General Hospital, Singapore; Changi General Hospital, Singapore; Singapore, Changi General Hospital, Singapore

## Abstract

**Objectives:** Sharp injuries are frequent occurrences in healthcare settings. According to the World Health Organization, >2 million occupational exposures to sharp injuries occur among 35 million healthcare workers (HCWs) annually. We report the 5-year incidence and trends of sharp injuries in an acute-care hospital. We compared the rates of injury, the distribution of injuries, and the type of exposure of HCWs during the prepandemic and pandemic eras. **Methods:** We conducted a retrospective analysis of a 5-year surveillance on self-reported sharp injuries in Changi General Hospital, a 1,000-bed acute-care hospital. The occupational groups, the type of sharps, and the incident activity involved were reviewed. The bloodborne pathogen statuses of the identified source patients were studied. **Results:** In total, 441 sharp injuries were reported from 2017 to 2021. Among the occupational groups, doctors reported the highest number of sharp injuries (N = 272, 61.7%), followed by nurses (N = 129, 29.3%) and other allied health professionals (N = 29, 6.4%). An increasing proportion of doctors reported sharp injuries from 2017 to 2019 (prepandemic era) and the proportion declined from 2020 to 2021 (pandemic era); 52 doctors (58.4%) reported sharp injuries in 2017, 61 (61.1%) in 2018, 72 (67.9%) in 2019, 47 (61%) in 2020, and 40 (57.1%) in 2021. Most sharp injuries were caused by solid sharps (212 of 441, 48.1%) and hollow-bore needles (205 of 441, 46.5%). Source patients were identified in 407 sharp injuries. From the known sources, 51 were seropositive: 20 for hepatitis B (HBV), 27 for hepatitis C (HCV), and 4 for human immunodeficiency virus (HIV). No seroconversion occurred. Overall, 198 sharp injuries (44.9%) were sustained during surgical procedures, 83 (18.8%) occurred during blood taking, and 44 (9.9%) occurred during injection administration. Also, 37.5% of sharp injuries among doctors occurred during surgical procedures, and 69.6% of sharp injuries in OT occurred among junior surgical doctors. **Conclusions:** The overall incidence of sharp injuries has decreased during the pandemic. Fewer elective surgical procedures were performed during the pandemic period. OT suturing training workshops and awareness programs on strategies for preventing sharps injuries in the operating theatre, targeted at surgical residents during the past 2 years, could have contributed to the decrease in the incidence of sharp injuries in our hospital. Sharp injuries pose a significant exposure to blood and body fluids and should be subject to continued epidemiological surveillance.

